# Correlation Analysis of Pathological Features and Axillary Lymph Node Metastasis in Patients with Invasive Breast Cancer

**DOI:** 10.1155/2022/7150304

**Published:** 2022-09-19

**Authors:** Hongye Chen, Xiangchao Meng, Xiaopeng Hao, Qiao Li, Lin Tian, Yue Qiu, Yuhui Chen

**Affiliations:** ^1^Department of General Surgery, The First Medical Center, Chinese PLA General Hospital, Beijing 100853, China; ^2^General Surgery, The First Hospital of Qinhuangdao, Qinhuangdao, 066600 Hebei, China; ^3^Department of Military Wounded Patient Management, The Eighth Medical Center of PLA General Hospital, Beijing 100091, China

## Abstract

**Objective:**

To investigate the risk factors of axillary lymph node metastasis in patients with invasive breast cancer.

**Methods:**

This study retrospectively included 122 cases of invasive breast cancer patients admitted to the First Medical Center of PLA General Hospital from January 2019 to September 2020. According to postoperative pathological results, axillary lymph node metastasis was divided into axillary lymph node metastasis (ALNM) group (*n* =40) and non-axillary lymph node metastasis (NALNM) group (*n* =82). General demographic information was collected and compared between the two groups. Collected pathological results included lymphovascular invasion (LVI) and the expression of estrogen receptor (ER), progestogen receptor (PR), human epidermal growth factor receptor 2 (HER-2), and Ki-67 detected by immunohistochemistry. Imaging parameters of dynamic contrast-enhanced magnetic resonance imaging (DCE-MRI) including apparent diffusion coefficient (ADC), early enhanced rate, and time-intensity curve (TIC) were also included into univariate analysis. The variables with differences between the two groups were compared by univariate analysis, and the related factors of axillary lymph node metastasis were analyzed by logistic regression model.

**Results:**

There was no significant difference in general demographic information between the two groups. No significant differences were found in the positive rates of HER-2, ER, PR, Ki-67, pathological types, and clavicular lymph node metastasis and skin chest wall invasion between the two groups (*P* > 0.05). The proportion of LVI in ALNM group was significantly higher than that in NALNM group (37.50% vs. 6.10%, *P* < 0.001). The proportion of breast cancer on the left side in the ALNM group was higher than that in the NALNM group, and the difference was statistically significant (70.00% vs. 47.56%, *P* = 0.019). There were no significant differences in the imaging parameters obtained by DCE-MRI between the two groups. Binary logistics regression analysis showed that LVI (OR =12.258, 95% CI =3.681-40.812, *P* < 0.001) and left breast cancer (OR =3.598, 95% CI =1.404-9.219, *P* = 0.008) were risk factors for axillary lymph node metastasis in patients with invasive breast cancer.

**Conclusion:**

The formation of vascular tumor thrombi in breast cancer tissue and left breast cancer are risk factors for axillary lymph node metastasis in invasive breast cancer and might be helpful for preoperative detailed assessment of the patient's condition.

## 1. Introduction

Breast cancer is one of the common female malignant tumors with high morbidity and mortality [1–3]. The occurrence and development of early invasive breast cancer were often accompanied by axillary lymph node metastasis and accurate axillary lymph node staging plays an important guiding role in the selection of local treatment plan, systemic comprehensive treatment decision, and prognosis judgment [4–6]. Currently, the gold standard for the diagnosis of axillary lymph node metastasis in breast cancer is axillary lymph node dissection and sentinel lymph node biopsy [7]. Features that are suggestive of axillary adenopathy may be seen with mammography, computed tomography (CT), and magnetic resonance imaging (MRI), but ultrasonography is the imaging modality of choice for evaluating axillary lymph nodes [8].

Breast cancer pathology molecules such as Ki-67, breast cancer molecular expression of estrogen receptor (ER), progesterone receptor (PR), and human epidermal growth factor receptor 2 (HER-2) are important markers to reflect the biological characteristics of breast tumor [9]. Lymphovascular invasion (LVI) is also an important indicator of poor prognosis in breast cancer patients [10]. Previous studies have made predictive model analysis on axillary lymph node metastasis from the aspects of imaging or tumor markers, including dynamic contrast-enhanced magnetic resonance imaging (DCE-MRI) and ultrasound [11]. However, it was also reported that patients with axillary sononegative breast cancer may have significant lymph node metastasis clinically [12]. In addition, there is still a lack of a prediction model including comprehensive factors of patients. Therefore, the purpose of this study was to explore the risk factors of axillary lymph node metastasis through retrospective analysis of the complete clinical medical records of patients with invasive breast cancer, so as to provide a basis for clinicians to choose surgical methods in the axillary region.

## 2. Materials and Methods

### 2.1. Study Population

This study retrospectively included 122 cases of invasive breast cancer patients admitted to the First Medical Center of PLA General Hospital from January 2019 to September 2020. Patients were divided into axillary lymph node metastasis (ALNM) group (*n* =40) and non-axillary lymph node metastasis (NALNM) group (*n* =82) according to the presence of axillary lymph node metastasis indicated by postoperative pathological results. Inclusion criteria: (1) patients who had received surgery and pathologically confirmed stage I-III invasive breast cancer; (2) prior to the operation, no special treatment such as chemoradiotherapy, endocrine therapy, and molecular targeted drug therapy was performed; (3) female patients aged between 18 and 65; (4) complete imaging data including X-ray, CT, and DCE-MRI; (5) complete clinical data.

Exclusion criteria: (1) carcinoma in situ; (2) combined history of upper axillary surgery, abdominal or thoracic surgery; (3) recurrent breast cancer; (4) complicated with other serious malignant tumors, autoimmune diseases, and serious damage to vital organs; (5) incomplete clinical data.

This study was approved by the ethics committee of the First Medical Center of PLA General Hospital (approval no. 2021-166) and the need for informed consent was waived due to the retrospective nature of the study.

### 2.2. Pathologic Analysis

The expression of ER, PR, HER-2, and Ki-67 was confirmed by immunohistochemistry. Antibodies and kits were purchased from Fuzhou Maixin Biotechnology Development Co., Ltd. Criteria for determining negative and positive molecular expression [13]: the expression of ER and PR was judged by the percentage of brownish yellow particles in the nucleus. The cancer cells with brown-yellow particles in the nucleus were identified as ER/PR positive cells. Five high magnification fields were randomly selected from each section to calculate the percentage of positive cells. When the percentage of positive cells was greater than 1%, it was identified as ER/PR positive cells.; HER-2 staining was performed according to the staining status of cell membrane: tumor cells with no staining at all or <10% were (-); >10% and cell membrane staining was (+); >10% and showing weak to moderately complete cell membrane staining was marked as (++); >10% showed strong and complete cell membrane staining as (+++). HER-2 (-) and (+) were judged as negative, (++) was unclear, and (+++) was positive. Tumors with HER-2 (++) were further confirmed by FISH test. FISH results were divided into positive, uncertain, and negative according to the HER-2 copy number or the ratio of HER-2 copy number to the centromeres of chromosome 17, and patients with uncertain FISH results were excluded; the diagnostic criteria for Ki-67 positive expression were the percentage of tumor nuclear staining in the hot field of the section, Ki-67 ≥ 14% as positive expression, Ki-67 <14% as negative expression. LVI was defined by the presence of tumor cells within lymphovascular spaces [14]. In HE staining samples, the phenomenon of flattened endothelial cells or lymphocytes lining the nests of tumor cells was regarded as lymphatic carcinoma thrombolus and the tumor cell nests surrounded by smooth muscle cells or accompanied by red blood cells were regarded as vascular tumor thrombus.

Pathologists observed the HE staining sections and immunohistochemical staining sections under light microscope and recorded the cell morphology of the lesions and the expressions of ER, PR, HER-2, and Ki-67 in paraffin sections after operation, so as to give the final diagnosis.

The histological classification of breast cancer is classified as invasive carcinoma of non-special type including invasive ductal carcinoma and invasive lobular carcinoma and invasive carcinoma of special type including medullary carcinoma and mucous adenocarcinoma [15].

### 2.3. MR Image Acquisition

The DCE-MRI was performed with 3.0 T MRI scanner (Simens Skyra 3.0 T). The patient took the prone position, and both mammary glands naturally hung in the groove of the mammary coil. Conventional three-dimensional (3D) positioning and correction scanning: axial plane T2-weighted imaging (T2WI) spectral adiabatic inversion recovery (T2WI-SPAIR) sequence scanning: relaxation time (TR) 4600 ms, echo time (TE) 98 ms, layer thickness 5.5 mm, interval 0 mm, matrix 320 × 256, field of view (FOV) 32 cm ×32 cm, excitation times 3, and layer number 24. Dynamic enhanced scanning: pre-scanning was performed first, and contrast agent GD-DTPA (Xi'an Ruixi Biotechnology Co., Ltd) was injected after satisfaction. The dosage was 0.1~0.2 mmol/kg, and the rate was 2 mL/s. After the injection, 20 mL of normal saline was injected into the flushing tube at the same rate, and dynamic enhanced scanning was performed at the same time. Transverse plane scanning parameters: TR of 4.1 ms, TE of 2.1 ms, slice thickness of 1.4 mm, interval of 0 mm, matrix of 320 ×320, FOV of 32 cm ×32 cm, excitation times of 0.71, and layer number of 150. The scan was performed immediately after the injection of the high-pressure syringe for 5 consecutive periods.

### 2.4. MR Image Interpretation

Two radiologists with more than 5 to 10 years of experience in breast imaging reviewed the MR images together and reached a consensus. The MRI morphological features of the lesions were analyzed and evaluated, including lesion location, shape, edge, T2WI signal, catheter dilation, and enhancement. Dynamic enhanced images were fed into SyngoMMWP post-processing workstation (Version VE40B) using Mean Curve software. The apparent diffusion coefficient (ADC) values were measured at the machine's accompanying post-processing workstation. The ADC values were calculated through the specific formula [16]: ADC = ln(SI1/SI2)/800 in which SI1 and SI2 were signal intensities for *b* =0 and 800 s/mm^2^. The time-intensity curve (TIC) of these lesions was divided into 3 types [17]: persistently enhancing, platform, and washout. The three-dimensional dimensions of the tumors were also recorded and compared.

### 2.5. Statistical Analysis

Statistical analysis was performed with SPSS 20.0 software (SPSS Inc., Chicago, IL, USA). Measurement data conforming to normal distribution were expressed as mean ± SD. Independent *t*-test was performed for parametric variables. Counting data were compared by chi-square test. Binary logistic regression analysis was performed to analyze the risk factors predictive for ALNM. *P* < 0.05 was considered statistically significant.

## 3. Results

### 3.1. Comparison of General Clinical Information

There were no statistically significant differences in age, smoking history, alcoholism history, family history, menopause, comorbidity, and body mass index (BMI) between the two groups (*P* > 0.05). The proportion of breast cancer on the left side in the ALNM group was higher than that in the NALNM group, and the difference was statistically significant (70.00% vs. 47.56%, *P* = 0.019) (Table 1).

### 3.2. Comparison of DCE-MRI Features

There was no statistical significance in the size of lesions between the two groups (*P* > 0.05) (Table 2). There were no significant differences in ADC, early enhancement rate, and TIC between the two groups (*P* > 0.05) (Table 3).

### 3.3. Comparison of Pathological Features

The proportion of LVI in ALNM group was significantly higher than that in NALNM group (37.50% vs. 6.10%, *P* < 0.001). There were no significant differences in the positive rates of HER-2, ER, PR, Ki-67, pathological types, clavicular lymph node metastasis, and skin chest wall invasion between the two groups (*P* > 0.05) (Table 4).

### 3.4. Logistic Regression Result

Binary logistics regression analysis showed that LVI (OR =12.258, 95% CI =3.681-40.812, *P* < 0.001) and left breast cancer (OR =3.598, 95% CI =1.404-9.219, *P* = 0.008) were risk factors for axillary lymph node metastasis in patients with invasive breast cancer (Table 5).

## 4. Discussion

Invasive breast cancer referred to breast cancer with the ability to invade surrounding tissue, lymph node metastasis, or distant metastasis [18–20]. This study investigated the risk factors for axillary lymph node metastasis in patients with invasive breast cancer, and the results suggested that vascular tumor plug and left breast cancer were the risk factors for axillary lymph node metastasis in patients with invasive breast cancer.

Breast cancer seriously affects women's life and health. Invasive breast cancer is a malignant tumor that has penetrated the basement membrane of mammary ducts or lobular acinus and invaded the stroma. The majority of invasive breast cancer is adenocarcinoma, which originates from mammary parenchymal epithelial cells, especially the ductal leaflet of the distal breast [21]. It was found that the occurrence and development of breast cancer were the result of synergistic action of multiple genes and factors [22]. Axillary lymph nodes are common metastatic sites of breast cancer. Axillary lymph node dissection is a common clinical operation, but this method has a lot of trauma and complications, and the excessive treatment of axillary lymph node negative patients does not improve the local control rate and long-term survival rate [23]. To clarify the rule of axillary lymph node metastasis and study the risk factors related to axillary lymph node metastasis of breast cancer are of positive significance for guiding clinical selection of reasonable surgical procedures, developing personalized treatment plans, and predicting the prognosis of patients. Therefore, finding biomarkers that can effectively predict axillary lymph node metastasis can provide new predictive indicators and therapeutic targets for breast cancer.

Currently, preoperative axillary evaluation of breast cancer patients is mainly carried out by imaging methods, among which ultrasound, molybdenum target, MRI, and CT are the most commonly used [24]. Routine breast MRI scans included short-tau inversion recovery (STIR) T2WI, diffusion-weighted imaging (DWI), and DCE-MRI sequences to reflect the morphological characteristics and functional status of tumors [25]. In clinical work, multiple sequences have been used to comprehensively determine the characteristics of tumors, especially DCE-MRI, which can reflect microangiogenesis and blood perfusion of tissues or lesions [26]. Dong, X et al. reported that the entropy of ROIs showed the best diagnostic ability to distinguish lymph node metastasis [27]. Mao, N et al. set a radiomics nomogram of DCE-MRI for the prediction of axillary lymph node metastasis. Their model revealed good calibration and discrimination with areas under the ROC curve (AUC) of 0.90 (95% CI, 0.85-0.95) [28]. Arefan, D et al. developed machine learning and found that the radiological features of segmented tumor areas in breast MRI were related to axillary lymph node status [29]. However, there was no statistical difference in MRI parameters between the axillary lymph node metastasis group and the non-metastasis group in this present study, which might be related to the small number of cases included in this study.

A variety of nomogram models related to breast cancer have been established, which are widely used in prediction of ALNM metastasis, showing high reference value for clinical decision-making. These columns include MSKCC Nomogram [30], Mayo Nomogram [31], and Cambridge Nomogram [32], which contain different predictive factors. Bevilacqua et al. [30] studied and published an MSKCC Nomogram for preoperative prediction of sentinel lymph nodes in 2007. In this study, 3786 patients who had undergone sentinel lymph node biopsy in the MSKCC database were retrospectively analyzed, and a multivariate prediction model was established. In this model, age, tumor size, tumor type, tumor location, vascular invasion, multifocal, histological grade, and ER and PR status were considered risk factors for ALNM. Subsequently, the author validated the model in 1548 patients, and the ROC curve was 0.754, showing good diagnostic value. Several studies reported that MSKCC Nomogram could provide reliable predictive analysis for assessing the risk of ALNM [33]. The present study suggested that LVI (OR =12.258, 95% CI =3.681-40.812, *P* < 0.001) and left breast cancer (OR =3.598, 95% CI=1.404-9.219, *P* = 0.008) were risk factors for axillary lymph node metastasis in patients with invasive breast cancer.

Multiple tumor markers have been used to diagnose and predict the prognosis of breast cancer [22, 34]. According to the hormone receptor (ER and PR) and HER-2 status, breast cancer was divided into three main subtypes: intracavity ER positive and PR positive, and then subdivided into intracavity A and B. HER-2 positive and three negative breast cancer [9]. Standardized diagnostic evaluation based on hormone receptors (ER and PR) and HER-2 was essential to determine these subtypes and the histochemical staining of proliferation marker protein Ki-67 (MKI67) could be used to differentiate intracavity A-like and B-like breast cancer [35]. Kustic et al. found that HER-2 was one of the adverse prognostic factors of breast cancer. When HER-2 was highly expressed, it was characterized by strong invasion and vigorous cell proliferation, suggesting the invasion and metastasis of breast cancer [36]. A previous study has recognized the value of LVI in breast cancer patients [14]. LVI could enhance breast cancer proliferation, which could lead to axillary lymph node metastasis [37]. In this study, logistic regression analysis suggested that LVI was one of the risk factors for axillary lymph node metastasis in patients with invasive breast cancer. In addition, this present study showed that patients with left invasive breast cancer were more likely to have axillary lymph node metastasis than those with right invasive breast cancer, which need further study.

There were also some limitations in this study. The first is the limited sample size of the study. Due to the retrospective nature of the study design, the sample size in this study was limited to inpatients. Secondly, the cases included in this study were from a single center, so there may be some bias in the selection of patients. Therefore, prospective studies with more scientific sample size and more comprehensive design are still needed to improve the quality of research results.

## 5. Conclusion

The formation of vascular tumor thrombi in breast cancer tissue and left breast cancer are risk factors for axillary lymph node metastasis in invasive breast cancer.

## Figures and Tables

**Table 1:** Comparison of general data between the two groups.

Index	ALNM group (*n* =40)	NALNM group (*n* =82)	*t*/*χ*^2^	*P* value
Age (year)	46.3 ± 10.5	48.8 ± 8.2	1.442	0.152^∗^
Affected side (*n*, %)			5.467	0.019^#^
Left side	28 (70.00%)	39 (47.56%)		
Right side	12 (30.00%)	44 (52.44%)		
Drinking history (*n*, %)	2 (5.00%)	0 (0.00%)	1.644	0.200^#^
Family history (*n*, %)	0 (0.00%)	3 (3.66%)	0.363	0.547^#^
Pausimenia (*n*, %)			0.881	0.348^#^
Yes	14 (35.00%)	36 (43.90%)		
No	26 (65.00%)	46 (56.10%)		
Hypertension (*n*, %)	2 (5.00%)	5 (6.10%)	0.029	0.865^#^
Diabetes mellitus (*n*, %)	1 (2.50%)	3 (3.66%)	0.042	0.838^#^
BMI (kg/m^2^)	24.9 ± 3.6	24.7 ± 3.7	0.306	0.760^∗^

ALNM: axillary lymph node metastasis; NALNM: non-axillary lymph node metastasis; BMI: body mass index; ^∗^, compared by *t*-test; ^#^, compared by chi-square test.

**Table 2:** Comparison of lesion size between the two groups.

Index	ALNM group (*n* =40)	NALNM group (*n* =82)	*t*	*P* value
X axis (cm)	2.27 ± 1.35	1.91 ± 0.96	1.692	0.093
Y axis (cm)	1.76 ± 1.21	1.52 ± 0.71	1.386	0.168
Z axis (cm)	1.30 ± 0.65	1.24 ± 0.56	0.501	0.618
Lesion volume (cm^3^)	11.90 ± 36.11	5.59 ± 9.34	1.490	0.139

ALNM: axillary lymph node metastasis; NALNM: non-axillary lymph node metastasis.

**Table 3:** Comparison of MRI parameters between the two groups.

Index	ALNM group (*n* =40)	NALNM group (*n* =82)	*t*/*χ*^2^	*P* value
ADC (10^−3^ mm^2^/s)	0.90 ± 0.16	0.89 ± 0.18	0.315	0.753^∗^
Early enhanced rate (*n*, %)			1.194	0.275^#^
>120	8 (20.00%)	24 (29.27%)		
≤120	32 (80.00%)	58 (70.73%)		
TIC category (*n*, %)			3.808	0.149^#^
Plateau	9 (22.50%)	25 (30.49%)		
Washout	28 (70.00%)	43 (52.44%)		
Persistently enhancing	3 (7.50%)	14 (17.07%)		

ALNM: axillary lymph node metastasis; NALNM: non-axillary lymph node metastasis; TIC: time-intensity curve; ADC: apparent diffusion coefficient; ^∗^, compared by *t*-test; ^#^, compared by chi-square test.

**Table 4:** Comparison of pathological features between two groups [cases, (%)].

Index	ALNM group (*n* =40)	NALNM group (*n* =82)	*χ* ^2^	*P* value
HER-2 (*n*, %)			2.325	0.508
(-)	6 (15.00%)	16 (19.51%)		
(1+)	7 (17.50%)	21 (25.61%)		
(2+)	19 (47.50%)	35 (42.68%)		
(3+)	8 (20.00%)	10 (12.20%)		
Ki-67 (*n*, %)			0.822	0.365
Positive	34 (85.00%)	64 (78.05%)		
Negative	6 (15.00%)	18 (21.95%)		
ER (*n*, %)			1.069	0.301
Positive	30 (75.00%)	68 (82.93%)		
Negative	10 (25.00%)	14 (17.07%)		
PR (*n*, %)			0.467	0.494
Positive	32 (80.00%)	61 (74.39%)		
Negative				
Supraclavicular LNM (*n*, %)	1 (2.50%)	0 (0.00%)	0.136	0.713
Invade the skin or chest wall (*n*, %)	1 (2.50%)	1 (1.22%)	0.056	0.813
Pathological types (*n*, %)			0.880	0.348
Special type	5 (12.50%)	6 (7.32%)		
Non-special type	35 (87.50%)	76 (92.68%)		
LVI (*n*, %)	15 (37.50%)	5 (6.10%)	19.343	0.000

ALNM: axillary lymph node metastasis; NALNM: non-axillary lymph node metastasis; HER-2: human epidermal growth factor receptor 2; ER: estrogen receptor; PR: progestogen receptor; LNM: lymph node metastasis; LVI: lymphovascular invasion.

**Table 5:** Logistic analysis of risk factors for axillary lymph node metastasis.

Index	*B*	Sig. (*P*)	OR	95% CI
LVI	2.506	0.000	12.258	3.681-40.812
Left side metastasis	1.280	0.008	3.598	1.404-9.219

LVI: lymphovascular invasion; CI: confidence interval.

## Data Availability

The datasets used and/or analyzed during the current study are available from the corresponding author upon reasonable request.
